# Investigating the Role of Ferroptosis-Related Genes in Ovarian Aging and the Potential for Nutritional Intervention

**DOI:** 10.3390/nu15112461

**Published:** 2023-05-25

**Authors:** Pei-Hsuan Lin, Wan-Ping Su, Chia-Jung Li, Li-Te Lin, Jim Jinn-Chyuan Sheu, Zhi-Hong Wen, Jiin-Tsuey Cheng, Kuan-Hao Tsui

**Affiliations:** 1 Department of Biological Sciences, National Sun Yat-sen University, Kaohsiung 804, Taiwan; peihsuan0308@gmail.com; 2 Department of Obstetrics and Gynaecology, Kaohsiung Veterans General Hospital, Kaohsiung 813, Taiwan; wanpeen@gmail.com (W.-P.S.); nigel6761@gmail.com (C.-J.L.); litelin1982@gmail.com (L.-T.L.); 3 Institute of Biomedical Sciences, National Sun Yat-sen University, Kaohsiung 804, Taiwan; sheu.jim@gmail.com; 4 Institute of Biopharmaceutical Sciences, National Sun Yat-sen University, Kaohsiung 804, Taiwan; 5 Department of Marine Biotechnology and Resources, National Sun Yat-sen University, Kaohsiung 804, Taiwan; wzh@mail.nsysu.edu.tw; 6 Department of Obstetrics and Gynaecology, National Yang-Ming University School of Medicine, Taipei 112, Taiwan; 7 Department of Obstetrics and Gynecology, Taipei Veterans General Hospital, Taipei 112, Taiwan; 8 Department of Medicine, Tri-Service General Hospital, National Defense Medical Center, Taipei 114, Taiwan

**Keywords:** nutrients, ferroptosis, ovarian aging

## Abstract

With advancing age, women experience irreversible deterioration in the quality of their oocytes, resulting in reduced fertility. To gain a deeper understanding of the influence of ferroptosis-related genes on ovarian aging, we employed a comprehensive approach encompassing spatial transcriptomics, single-cell RNA sequencing, human ovarian pathology, and clinical biopsy. This investigation revealed the intricate interactions between ferroptosis and cellular energy metabolism in aging germ cells, shedding light on the underlying mechanisms. Our study involved 75 patients with ovarian senescence insufficiency, and we utilized multi-histological predictions of ferroptosis-related genes. Following a two-month supplementation period with DHEA, Ubiquinol CoQ10, and Cleo-20 T3, we examined the changes in hub genes. Our results showed that TFRC, NCOA4, and SLC3A2 were significantly reduced and GPX4 was increased in the supplement group, confirming our prediction based on multi-omic analysis. Our hypothesis is that supplementation would enhance the mitochondrial tricarboxylic acid cycle (TCA) or electron transport chain (ETC), resulting in increased levels of the antioxidant enzyme GPX4, reduced lipid peroxide accumulation, and reduced ferroptosis. Overall, our results suggest that supplementation interventions have a notable positive impact on in vitro fertilization (IVF) outcomes in aging cells by improving metal ion and energy metabolism, thereby enhancing oocyte quality in older women.

## 1. Introduction

The ovary is a critical organ for maintaining female reproductive and endocrine functions and undergoes aging earlier and faster than other organs [1]. Ovarian aging is characterized by a progressive decline in oocyte quantity and quality, and its regulation mechanism involves various physiological changes, including mitochondrial dysfunction, metabolic disturbance, aneuploidy, and epigenetic modifications. Mitochondrial dysfunction leads to reduced energy production critical for cell growth and division. Additionally, metabolic activity and gene expression changes can have negative effects. Older women have decreased levels of glutathione peroxidase and superoxide dismutase in follicles, suggesting that oxidative stress and lipid peroxides cannot be efficiently cleared in senescent cells [2]. Oxidative stress causes cellular damage and accelerates the aging process [3]. Therefore, reducing lipid peroxides, scavenging free radicals, and increasing mitochondrial respiration are promising strategies for improving ovarian hypofunction [4]. Several treatments and clinical interventions aim to achieve these goals, but more research is needed to develop effective treatments.

Ferroptosis is a unique type of programmed cell death that is characterized by iron-dependent lipid peroxidation, resulting in the accumulation of toxic lipid hydroperoxides and ultimately leading to cell death [5]. Transferrin receptor (TFRC) is a key protein involved in cellular iron uptake and is essential for cell growth [6]. The receptor functions by transporting iron from the extracellular environment into the cell via receptor-mediated endocytosis. The extracellular domain of TFRC has a high affinity for di-iron transferrin and is critical for proper iron uptake by cells. Glutathione peroxidase 4 (GPX4) is a selenoenzyme that plays a crucial role in preventing ferroptosis by reducing lipid hydroperoxides to nontoxic lipid alcohols using glutathione (GSH) [7,8]. The cystine-glutamate antiporter system, composed of light chain subunit SLC7A11 and heavy chain subunit SLC3A2, is another key regulator of ferroptosis that can be inhibited by Erastin. Inhibition of this system leads to a reduction in cystine intake and can also trigger ferroptosis [9].

Ferritin, a major intracellular iron storage protein, is a critical component of the antioxidant defense system in cells [6]. Ferritin autophagy is regulated by nuclear receptor coactivator 4 (NCOA4), a recently identified autophagy cargo receptor. NCOA4 is responsible for mediating ferritin phagocytosis, a critical step in iron metabolism and storage [10]. By chelating redox-active iron, ferritin plays an important role in protecting cells from oxidative stress. However, ferritin phagocytosis has also been shown to be involved in ferroptosis [8]. Knockdown of NCOA4 inhibits ferritin phagocytosis and reduces the amount of bioavailable intracellular labile iron pools, which can block lipid peroxidation and ferroptosis [11]. Overall, understanding the regulation of ferroptosis is crucial for developing new therapeutic strategies for various diseases, including cancer, neurodegenerative disorders, and ischemic injuries.

**Figure 1:**
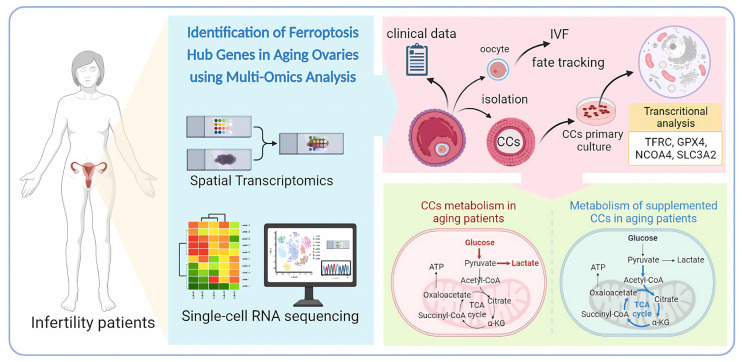
Schematic representation of supplementation improving energy metabolism through inhibition of ferroptosis in ovarian aging patients.

The objective of this study was to explore the potential benefits of nutritional supplements on aging germ cells. The first step was to utilize spatial transcriptomics and single-cell RNA sequencing to screen for potential targets. This involved analyzing the gene expression patterns of individual cells to identify genes that could be targeted for intervention. The study also investigated the upstream transcription factors that regulate these ferroptosis-related genes. Subsequently, the effects of a combination of nutritional supplements on these genes were examined. The study employed a multi-omics approach to screen for nutritional supplements and evaluated their efficacy in patients with ovarian aging. Additionally, the effects of the supplements on cellular energy metabolism were investigated to determine their ability to reduce cellular damage caused by aging (Figure 1).

## 2. Materials and Methods

### 2.1. Spatial Transcriptomics (ST) Analysis

In this study, we investigated the expression levels and spatial distribution of TFRC, GPX4, NCOA4, and SLC3A2 in tissue sections using ST data obtained from a previous study (GSE188257). To achieve this, we aggregated UMIs in each bin100 defined point and then performed clustering of similar ST points and dimensionality reduction using RunPCA, FindNeighbors, and FindClusters functions. Cluster annotation was performed based on H&E sections, and cell markers were used for further annotation. The ssGSEA algorithm was utilized to score common cell types based on the average expression matrix of different clusters, as some clusters exhibited high expression of multiple cell markers. This method has been found to be more effective in ST. Spatial transcriptomics offers valuable insights into the spatial distribution and expression patterns of crucial genes within tissue sections, providing valuable information for future investigations into underlying biological processes and mechanisms.

### 2.2. Single-Cell RNA Sequencing Analysis

The transcriptomic data for individual cells were obtained from three publicly available databases, namely GSE143380, GSE118127, and GSE146512, hosted by the GEO database. Quality control was carried out using the R software Seurat, which involved filtering out genes expressed in fewer than three cells and genes with mitochondrial gene expression of more than 10%. Additionally, only cells expressing at least 200 genes were selected to minimize gene expression fluctuations caused by single-cell and batch effects while retaining as much gene expression information as possible. The UMAP clustering algorithm was applied using the “BiocManager” and “GSVA” packages in R programming language to analyze the data. The “SingleR” package was used to compare the known cellular marker genes and label each subpopulation with its corresponding cell type.

### 2.3. Ethics Statem

The Institutional Review Board of the Kaohsiung Veterans General Hospital (KSVGH21-CT1-43) approved all procedures performed in this study. The study was conducted in compliance with the principles outlined in the Declaration of Helsinki.

### 2.4. Clinical Biopsies and Collection

**Figure 2:**
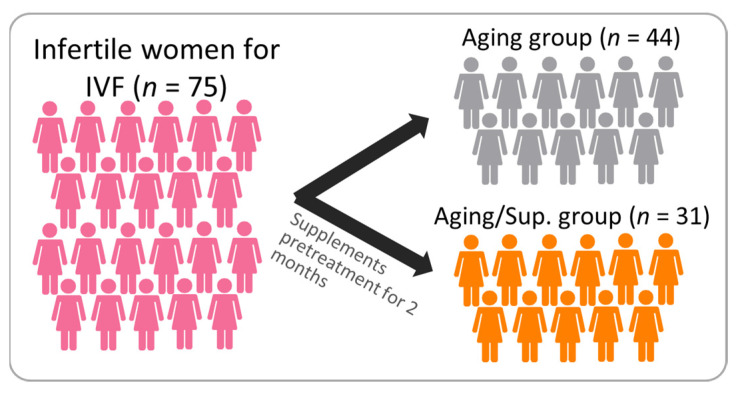
Flowchart illustrating study design and eligible study selection.

In May 2021, the IVF Center of Kaohsiung Veterans General Hospital conducted this study and recruited participants. The study groups included infertile patients categorized into the aging group and the aging-taking supplements group (aging/Sup.). The supplement group took DHEA capsules (New Dios-25 Veg capsules Toppure Biotechnology Co., Ltd., Taiwan), Ubiquinol CoQ10 Capsules (Circuform Softgel Capsules Toppure Biotechnology Co., Ltd., Taiwan), and Cleo-20 T3 soft capsules (Supremevitamin Biotechnology, Co., Ltd., Taiwan) daily for at least eight weeks before undergoing in vitro fertilization (IVF) (Figure 2). Patients with a history of oophorectomy, previous donor cycles, pelvic radiotherapy or chemotherapy, and hormone therapy within the past three months were excluded from the study. Table 1 and Table 2 show the clinical characteristics of the participants. The study was conducted in compliance with the Declaration of Helsinki by the World Medical Association, and written informed consent was obtained from all the participants.

### 2.5. Acquiring Cumulus Cells from Patients

The CCs were collected from patients using previously published methods [12]. The procedure involved separating the CCs from the oocytes, mechanically disassembling them, and pooling them together. The pooled CCs were then washed multiple times in a PBS/BSA solution using a series of centrifugation steps at 800× *g* for 5 min. The final precipitate was resuspended in Histopaque 1077, along with fetal bovine serum, insulin, transferrin, sodium selenite, and androstenedione. The CCs were then cultured on a multi-plate and kept in a humidified incubator with 5% CO_2_ at 37.5 °C for 24 h for further analysis.

### 2.6. RNA Extraction and Real-Time Polymerase Chain Reaction (PCR)

Total RNA was extracted from patient cells using the EasyPrep Total RNA Kit to identify any changes in the transcriptome. The ToolScript MMLV RT Kit was used to prepare cDNA from the mRNA samples. To perform qPCR, TOOLS 2× SYBR qPCR Mix was used for all cDNAs. These reagents were obtained from BIOTOOLS Co., Ltd., located in Taipei, Taiwan.

### 2.7. Statistical Analysis

The measurements in this study were independently conducted at least four times, and the results presented are the mean ± standard error of the mean (S.E.M) of multiple measurements. Statistical significance was determined using GraphPad Prism 8.0 (GraphPad Software, San Diego, CA, USA) and Tukey post-hoc tests were applied after performing a two-way analysis of variance to compare the means between groups. A *p*-value of less than 0.05 was considered statistically significant.

## 3. Results

### 3.1. Demographic and Clinical Characteristics of Patients Undergoing In Vitro Fertilization (IVF) Cycles

In this study, 75 patients were enrolled from the IVF Center of Kaohsiung Veterans General Hospital and divided into two groups: aging (n = 44) and aging/Sup. (n = 31). Table 1 presents the baseline characteristics of both groups, and the analysis revealed a significant difference in mean basal follicle-stimulating hormone (FSH) levels between the two groups. The aging/Sup. group had lower FSH levels (5.7 ± 4.7) than the aging group (8.1 ± 9.6). However, no significant differences were observed in other baseline characteristics such as age, body mass index (BMI), duration of infertility, primary infertility, secondary infertility, basal estradiol (E2), and basal luteinizing hormone (LH) levels.

### 3.2. Clinical and Cycle Characteristics of IVF Patients with Aging to Stimulation and Taking Supplements

The study analyzed the patient characteristics and pregnancy outcomes of IVF cycles in the aging and aging/Sup. groups, presented in Table 2. The results showed no significant differences in the duration of stimulation and the dose of gonadotropin between the two groups. However, when comparing the clinical characteristics of the aging/Sup. group with the aging group, a significant difference was observed in the number of retrieved oocytes (14.9 ± 7.1 vs. 6.8 ± 4.9), metaphase II oocytes (12.0 ± 5.8 vs. 5.1 ± 3.3), fertilized oocytes (9.9 ± 4.9 vs. 7.6 ± 2.7), number of day three (D3) embryos (9.1 ± 5.0 vs. 4.1 ± 2.7), and the number of top-quality D3 embryos (3.4 ± 2.5 vs. 1.9 ± 1.5). These results suggest that the use of supplements may have a positive effect on ovarian response and oocyte quality in patients with poor ovarian reserve.

### 3.3. Analysis of Spatial Transcriptomics and Single-Cell RNA Sequencing Databases to Evaluate Ferroptosis-Related Genes

**Figure 3:**
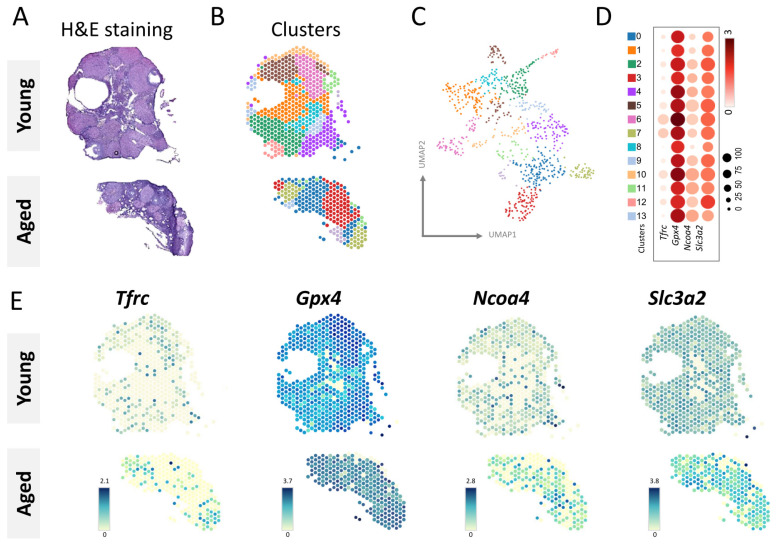
Gene expression of spatial transcriptome-defined clusters in young and aging mouse ovaries. (**A**) By using spatial transcriptomics, tissue sections were analyzed to identify clusters, and the alignment with morphology was confirmed by hematoxylin and eosin staining and cluster mapping. (**B**) Thirteen unsupervised clusters of spatial transcription histology were overlaid on ovarian samples. (**C**) A UMAP projection of the spatial transcription histology data containing the 13 unsupervised clusters defined by Space Ranger was generated. (**D**) Gene expression levels in different clusters were presented using dot plots. (**E**) Visium spatial gene expression was used to show the spatial location and gene variation of Tfrc, Gpx4, Ncoa4, and Slc3a2 in different tissue sections.

In this study, we utilized spatial transcriptomics techniques to map transcriptomic signatures directly onto histological images of mouse ovaries (GSE188257) stained with H&E (Figure 3A). We analyzed a total of 4263 spots and 32,285 genes measured, generating 13 unsupervised clusters with Space Ranger (Figure 3B). The Uniform Manifold Approximation and Projection (UMAP) of these clusters obtained with Space Ranger are shown in Figure 3C. The dot plots in Figure 3D depict the normalized, log-transformed, and variance-scaled expression of various cell clusters (y-axis) and characteristic genes (x-axis) in the data. We analyzed the expression levels of ferroptosis hub genes and found that Tfrc, Ncoa4, and Slc3a2 were expressed at higher levels in the aging group compared to the young group, while Gpx4 showed higher expression in the young group (Figure 3E).

**Figure 4:**
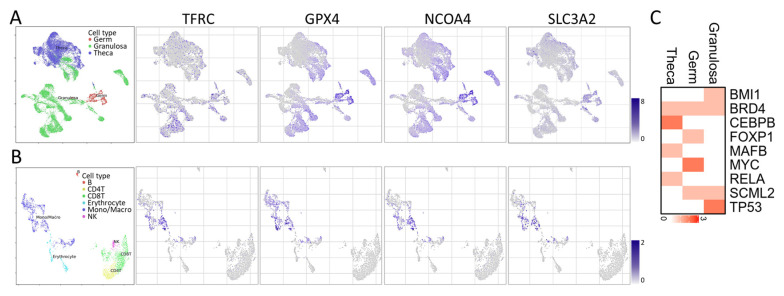
Single-cell RNA-sequencing analysis enables the identification of specific cells within human ovarian datasets. (**A**) shows the relative proportions of the three cell types found in public datasets and the distribution and changes of genes in the database. (**B**) Expression levels and distribution of four genes in different immune cells. (**C**) Three potential transcription factors upstream of germ cells.

**Figure 5:**
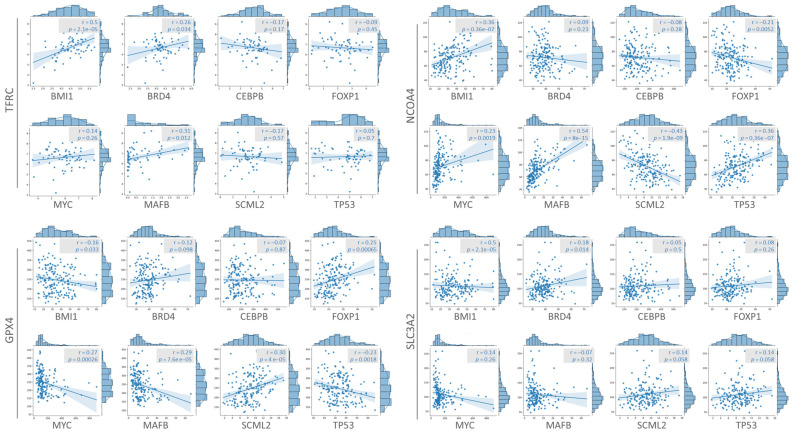
Association of TFRC, GPX4, NCOA4, and SLC3A2 with potential transcription factors.

To investigate the transcriptome of ferroptosis-related genes in human aging ovaries and to examine the heterogeneity of various cell types in the ovarian microenvironment, we utilized three databases (GSE143380, GSE118127, and GSE146512) containing 12 cell types and 22 clusters, which were derived from three ovarian samples (Figure 4A). Following the removal of batch effects and quality control, we analyzed a total of 86,161 cells and identified 22 significant cell clusters using UMAP plots. The analysis was initially focused on three major cell clusters of ovarian tissue, namely Germ, Granulosa, and Theca cells, revealing that a high proportion of the four ferroptosis-related genes were expressed in granulosa cells. Upon further subgrouping of different immune cells, most of the four genes were found to be associated with macrophage regulation (Figure 4B). Additionally, we analyzed the potential upstream transcription factor regulators and found higher levels of BMI1, BRD4, and TP53 in granulosa cells based on the heatmap (Figure 4C). Upon careful analysis, we observed statistically significant correlations between TFRC, GPX4, NCOA4, and SLC3A2 with the eight identified potential upstream transcription factors, as depicted in Figure 5. These findings suggest a potential regulatory relationship between these transcription factors and the expression of TFRC, GPX4, NCOA4, and SLC3A2 genes.

### 3.4. Modulation of Ferroptosis and Energy Metabolism Pathway Shifts in Aging Patients by Supplementation

**Figure 6:**
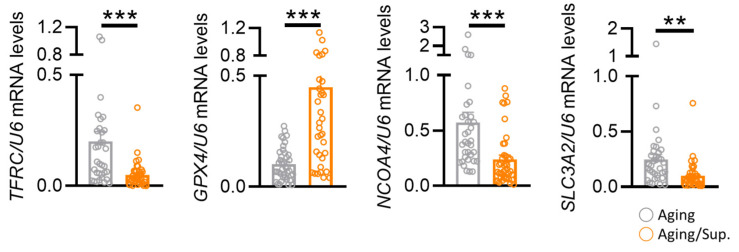
Differential expression of ferroptosis-related genes in human biopsies. Changes in human granulosa cell ferroptosis-related genes assessed by mRNA expression levels. ** *p* < 0.01, *** *p* < 0.001.

Next, we explored whether the supplementation could induce alterations in ferroptosis-related genes and remodel metabolic pathways. We investigated the association of nutritional epigenomics with these four genes by recruiting aging infertility patients for genetic validation. Nutritional supplements were selected based on the potential upstream transcription factors, including DHEA, CoQ10, and T3, which have been reported to have potential functions in regulating these genes. We tested whether these genes were improved by the nutritional supplements and analyzed the changes in the levels of these genes in the granulosa cells isolated from the oocytes of the patients. Our results showed that TFRC, NCOA4, and SLC3A2 were significantly reduced, and GPX4 was significantly increased in aging patients taking nutritional supplements (Figure 6). 

**Figure 7:**
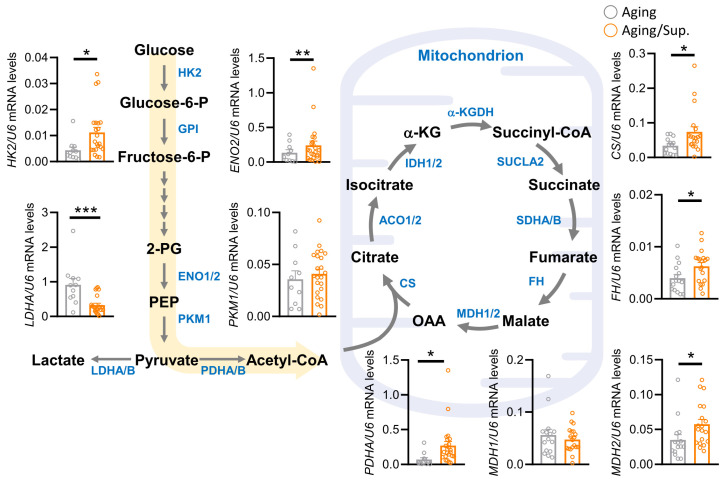
Supplementation affects metabolic reprogramming of cumulus cells in aging ovarian patients. Metabolic pathway and metabolite level diagrams in the glycolysis and TCA cycle pathways. * *p* < 0.05 and ** *p* < 0.01, *** *p* < 0.001.

We further analyzed the molecular mechanisms of energy metabolism genes in cumulus cells from both supplemented and un-supplemented aging patients. Our results showed significant differences in metabolites from glycolysis and the TCA cycle between the two groups. In the aging/Sup. group, we observed increases in hexokinase 2 (HK2) and enolase 2 (ENO2), indicating higher glucose consumption, while lactate dehydrogenase A (LDHA) showed decreases, indicating less lactate. Additionally, the expression levels of key genes regulating the TCA cycle, such as citrate synthase (CS), fumarate hydratase (FH), and malate dehydrogenase 2 (MDH2), were significantly increased in aging/Sup (Figure 7). These findings suggest that aging/Sup. induces changes in glucose metabolism and TCA cycle in cumulus cells from aging patients, which may contribute to enhanced cellular energy production and improved IVF cycle outcomes. Overall, our study sheds light on the potential benefits of metabolic reprogramming through supplementation in improving IVF success rates in aging patients.

## 4. Discussion

Senescent cells are characterized by an accumulation of iron caused by impaired lysosomal ferritin phagocytosis, which leads to an excess of redox-active iron when occurring in mitochondria [13]. This iron overload is believed to be a contributor to aging-related oxidative damage and can result in various forms of programmed cell death, including mitophagy, apoptosis, and ferroptosis [14]. Moreover, chronic iron overload has been shown to upregulate the expression of inducible nitric oxide synthase (iNOS) and enhance the binding of NF-kB to the iNOS gene promoter [15].

We examined the impact of ion concentration accumulation on gene regulation in the ovary using spatial transcriptome data. Our findings revealed distinct differences in ferroptosis-related genes between aged and young dams. Subsequently, we plan to conduct clinical trials employing nutritional genomics to analyze the correlation between ferroptosis-related genes and supplements. However, it should be noted that previous studies have observed hemosiderin and lipofuscin as markers of aging in mouse ovaries, indicating inflammatory senescence associated with ovarian aging [16,17,18]. Iron overload affects ovarian aging beyond the upregulation of ferroptosis-related genes, as evidenced by studies demonstrating the antiproliferative and apoptosis-inducing activities of EGR1 on granulosa cells during follicular atresia in aging mouse ovaries, as well as the impact of chronic low-grade systemic inflammation induced by the NLRP3 inflammasome on follicle reduction in aging ovaries [12,19]. In a mouse model of DHEA-induced polycystic ovary syndrome, iron salts were found to stimulate TFRC, increase iron levels, and trigger various forms of programmed cell death [20]. Hence, the effects of iron overload on ovarian aging are multifaceted.

In this research, we administered three types of nutritional supplements to patients with aging ovaries: dehydroepiandrosterone (DHEA), Coenzyme Q10 (Q10), and D3 (T3). DHEA is a steroid that is produced by the adrenal cortex reticular formation and the follicular membrane cells of the ovary. It plays a critical role in the formation of androgens such as androstenedione (A), testosterone (T), and estradiol (E2), which are essential hormones for women [21,22]. Q10 is a key element in mitochondrial activity, as mutations in the enzyme responsible for Q10 synthesis lead to phenotypes involving high energy-consuming tissues, such as the central nervous system, skeletal muscle, and kidney [23,24]. In humans, Q10 concentrations in some tissues start to decline after the age of 30 [25,26], which may contribute to the aging process. The timing of the age-related decline in Q10 availability appears to coincide with a decline in fertility and an increase in embryonic aneuploidy. In fact, previous studies have reported a correlation between low plasma Q10 levels and spontaneous abortion [27]. In addition, the levels of Q10 in follicular fluid are associated with oocyte maturation and embryonic grade during in vitro fertilization [28]. These findings suggest that the oocyte is a useful target for Q10 supplementation, but the granulosa/oocyte and/or uterine environment may also benefit, contributing to the enhanced fertility of females treated with Q10. Furthermore, our research showed that TP53 has control over the genes related to the regulation of iron death, as depicted in Figure 3C. Hence, we attempted to target P53, and D3 has been previously reported to prevent cardiac ischemia/reperfusion mitochondrial injury and cell loss by regulating the miR30a/p53 axis [29,30]. Since the heart is also a mitochondria-rich and energy-intensive organ, we offer oral D3 to activate P53 and mitochondrial function in patients with aging ovaries, which may be beneficial for their overall health.

Our research group has been focusing on the molecular regulation of mitochondria in aging granulosa and cumulus cells and seeking nutritional supplements that can effectively improve the fertility of patients. In this study, we discovered that the use of supplements can reprogram metabolic pathways, leading to the activation of mitochondrial oxidative phosphorylation. Our findings are consistent with previous studies that have demonstrated the protective effects of DHEA on human granulosa cells by preventing apoptosis and necroptosis and on cumulus cells by altering energy metabolism [31,32,33]. In addition, we have previously used Q10 to improve oxeiptosis caused by aging [34]. It has been reported that DHEA activates CREB1, a key transcription factor for energy metabolism, and regulates downstream genes such as PGC1a and TFAM, which are involved in biosynthesis [33]. Therefore, DHEA may regulate transcription factors that affect energy metabolism pathways in cells, leading to the reduction of programmed cell death caused by aging, such as mitophagy, necrosis, apoptosis, and ferroptosis, as mentioned in this study [35,36,37]. However, it is important to note that our study has a limitation of a small sample size, which requires a cautious approach to the interpretation of the data.

## 5. Conclusions

In this study, we employ an innovative spatial transcriptional and single-cell sequencing methodology to explore the influence of ferroptosis-related genes on reactive oxygen species (ROS) regulation during ovarian aging. Our findings indicate that targeting specific upregulated ferroptosis-associated genes through supplementation leads to a reduction in their expression levels, resulting in altered glycolysis and enhanced mitochondrial oxidative phosphorylation. This improved energy metabolism in senescent cells correlates with increased pregnancy rates among infertile patients, as depicted in Figure 1.

## Figures and Tables

**Table 1:** Basic characteristics of patients in the aging and aging/Sup. groups.

Parameters	Aging (n = 44)	Aging/Sup. (n = 31)
Age (years)	39.1 ± 4.9	38.7 ± 4.4
BMI (kg/m^2^)	23.3 ± 4.5	22.8 ± 3.0
Duration of infertility (years)	4.2 ± 3.3	3.9 ± 3.3
Previous IVF failure (n)	1.1 ± 1.3	0.7 ± 1.0
Types of infertility n (%)		
Primary infertility	19/44 (43%)	16/31 (52%)
Secondary infertility	25/44 (57%)	15/31 (48%)
Basal FSH (IU/L)	8.1 ± 9.6	5.7 ± 4.7 **
Basal E2 (pg/mL)	117.8 ± 100.8	113.3 ± 103.6
Basal LH (IU/L)	6.4 ± 6.8	5.0 ± 2.8

IVF, in vitro fertilization; FSH, follicle stimulation hormone; E2; Estradiol; LH, luteinizing hormone. ** *p* < 0.01.

**Table 2:** Cycle characteristics and pregnancy outcome in the aging and aging/Sup. groups.

Parameters	Aging (n = 44)	Aging/Sup. (n = 31)
Stimulation duration (days)	10.7 ± 2.8	10.6 ± 1.4
No. of oocytes retrieved (n)	6.8 ± 4.9	14.9 ± 7.1 ***
No. of metaphase II oocytes (n)	5.1 ± 3.3	12.0 ± 5.8 ***
Maturation rate (%)	79.6 ± 19.7	80.9 ± 11.1
No. of fertilized oocytes (n)	4.3 ± 2.9	9.9 ± 4.9 ***
Fertilization rate (%)	83.4 ± 22.7	83.7 ± 14.4
No. of Day 3 embryos (n)	4.1 ± 2.7	9.1 ± 5.0 ***
No. of top-quality D3 embryos (n)	1.9 ± 1.5	3.4 ± 2.5 **

** *p* < 0.01, *** *p* < 0.001.

## Data Availability

Not applicable.
